# Rapid response infrastructure for pandemic preparedness in a tertiary care hospital: lessons learned from the COVID-19 outbreak in Cologne, Germany, February to March 2020

**DOI:** 10.2807/1560-7917.ES.2020.25.21.2000531

**Published:** 2020-05-28

**Authors:** Max Augustin, Philipp Schommers, Isabelle Suárez, Philipp Koehler, Henning Gruell, Florian Klein, Christian Maurer, Petra Langerbeins, Vanessa Priesner, Kirsten Schmidt-Hellerau, Jakob J Malin, Melanie Stecher, Norma Jung, Gerhard Wiesmüller, Arne Meissner, Janine Zweigner, Georg Langebartels, Felix Kolibay, Victor Suárez, Volker Burst, Philippe Valentin, Dirk Schedler, Oliver A Cornely, Michael Hallek, Gerd Fätkenheuer, Jan Rybniker, Clara Lehmann

**Affiliations:** 1 University of Cologne, Department I of Internal Medicine, Division of Infectious Diseases, Cologne, Germany; 2 German Center for Infection Research (DZIF), Partner Site Bonn-Cologne, Cologne, Germany; 3 University of Cologne, Center for Molecular Medicine Cologne, Cologne, Germany; 4 These authors contributed equally to this article; 5 Institute of Virology, Faculty of Medicine and University Hospital Cologne, University of Cologne, Cologne, Germany; 6 Cologne Excellence Cluster on Cellular Stress Responses in Aging-Associated Diseases (CECAD), Department I of Internal Medicine, Clinical Trials Centre Cologne (ZKS Köln), University of Cologne, Cologne, Germany; 7 Department I of Internal Medicine and Center of Integrated Oncology Aachen, Bonn, Köln, Düsseldorf, University of Cologne, Cologne, Germany; 8 Public Health Department Cologne, Cologne, Germany; 9 Department of Hospital Hygiene and Infection Control, University Hospital Cologne, Cologne, Germany; 10 Department for Clinical Affairs, University of Cologne, Germany; 11 Department II of Internal Medicine (Nephrology, Rheumatology, Diabetes, and General Internal Medicine) and Center for Molecular Medicine Cologne, University of Cologne, Faculty of Medicine and University Hospital Cologne, Cologne, Germany; 12 University of Cologne, Medical Faculty and University Hospital Cologne, Department of Anaesthesiology and Intensive Care Medicine, Cologne, Germany

**Keywords:** COVID-19, pandemic preparedness, SARS-CoV-2, risk assessment, outbreak

## Abstract

The coronavirus disease (COVID-19) pandemic has caused tremendous pressure on hospital infrastructures such as emergency rooms (ER) and outpatient departments. To avoid malfunctioning of critical services because of large numbers of potentially infected patients seeking consultation, we established a COVID-19 rapid response infrastructure (CRRI), which instantly restored ER functionality. The CRRI was also used for testing of hospital personnel, provided epidemiological data and was a highly effective response to increasing numbers of suspected COVID-19 cases.

The coronavirus disease (COVID-19) pandemic caused by the severe acute respiratory syndrome coronavirus-2 (SARS-CoV-2), is a major public health emergency with high case fatality in the elderly population and in patients with co-morbidities [1-3]. Shortly after the occurrence of the first cases in North-Rhine Westphalia, Germany on 25 February 2020 [4], staff of the emergency room (ER) at the University Hospital Cologne (UHC) were largely occupied with managing suspected COVID-19 cases presenting with mostly mild or no symptoms, which hampered rational care of other patients.

We here describe the implementation of a COVID-19 rapid response infrastructure (CRRI), which allowed the UHC to maintain full functionality in spite of well over 1,000 suspected COVID-19 cases consulting the CRRI in the first 2 weeks of the epidemic in Germany.

## COVID-19 rapid response infrastructure

**Figure 1:**
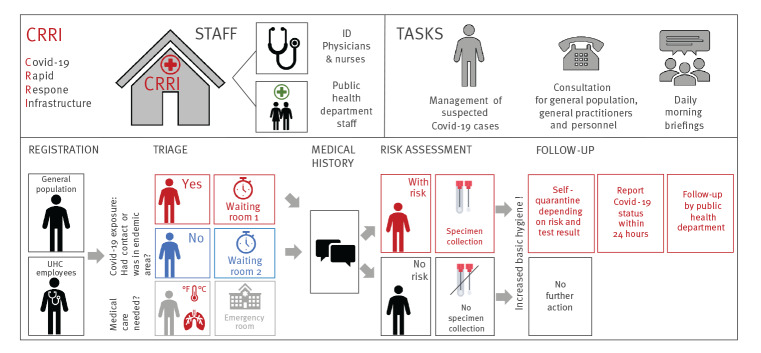
COVID-19 rapid response infrastructure at University Hospital Cologne, North Rhine Westphalia, Germany, February 2020

COVID-19: coronavirus disease; CRRI: COVID-19 rapid response infrastructure; ID: infectious disease; UHC: University Hospital Cologne.

On 26 February 2020, the UHC hospital board decided to set up an infrastructure to reduce the workload in the ER. Within 24 hours, a decommissioned stand-alone building at UHC was selected, reactivated and fully fitted out with IT systems and medical equipment, providing working space for medical personnel (five doctors and four nurses) and at least two representatives of the Cologne public health department (Figure 1). Sign posts led the patients who considered they may have COVID-19 directly to the CRRI in order to prevent the ER from being overcrowded. Upon registration, patients were equipped with face masks and underwent a triage.

In order to manage increasing numbers of suspected COVID-19 cases among patients and personnel, the CRRI was implemented. By means of basic triage, risk-based stratification as well as risk assessment, the indication of (i) naso-/oropharyngeal swab (ii) self-quarantine and (iii) report to public health department on site, was evaluated.

### Triage and patient management

First, suspected COVID-19 cases presenting with fever > 38.5 °C, chest pain and/or severe dyspnoea who might necessitate hospital admission were redirected to the ER as the CRRI was not equipped for an in-depth medical consultation (e.g. X-ray and electrocardiogram). Second, patients with a risk of SARS-CoV-2 exposure were placed in two separate waiting rooms according to risk-based stratification. Third, asymptomatic and symptomatic patients with no known SARS-CoV-2 exposure were clinically assessed, and, if not severely ill, referred to outpatient treatment at their general practitioner or their home environment (Figure 1).

According to recommendations by the German national public health institute, the Robert Koch Institute (RKI), a nasopharyngeal swab was taken from all symptomatic patients at risk of SARS-CoV-2 infection [5]. At the end of February, being at risk of a SARS-CoV-2 infection was defined as either (i) contact to a laboratory-confirmed COVID-19 case within the last 14 days or (ii) stay in a COVID-19 risk area within the last 14 days [5]. At implementation of the CRRI, solely regions of (i) Italy (Lodi Province and Vo City), (ii) South Korea (Gyeongsangbuk-do Province), (iii) Iran (Ghom Province) and (iv) China (Hubei Province including Wuhan City and Wengzhou City, Hangzhou City, Ningbo City, Taizhou City) were defined as risk areas [6]. Later on, new risk areas were added (e.g. all of Italy and Austrian Tyrol).

Staff of the Cologne public health department were physically present and interviewed all patients after the infectious disease (ID) physicians had assessed the patients clinically. After swabs were taken from suspected COVID-19 cases [5], official notification according to the German Protection Against Infection Act was carried out on site, so that no delay occurred. This enabled contact tracing as soon as the laboratory results were available, mostly within 24 hours after the swab was taken.

Symptomatic patients at risk were requested to self-quarantine until COVID-19 status was communicated to them via phone. Subsequently, the continuation and duration of self-quarantine was assessed by the public health department depending on (i) SARS-CoV-2 status and (ii) individual risk.

After the occurrence of the first SARS-CoV-2 infections among hospital staff, a second unit with a fast-track testing lane was established within the CRRI for UHC employees in order to separate them from the general population and to ensure a timely diagnostic service with the aim to maintain medical infrastructure. In contrast to the general population, both asymptomatic and symptomatic employees of the hospital were requested via the hospital intranet to approach the CRRI if they had contact to a SARS-CoV-2 positive person or returned from a COVID-19 risk area within the last 14 days.

### Patient documentation and diagnostics

Number of consultations per day and patient characteristics were collected from 27 February to 12 March 2020, and compared to the patient visits in the ER during the identical time period in 2019 (27 February to 12 March). All patients seeking SARS-CoV-2 testing were seen by a trained ID physician who documented the symptoms as reported by the patients.

Nasopharyngeal swabs were placed in universal transport medium (UTM) (Copan Diagnostics, Murrieta, United States (US)) and nucleic acids were extracted from UTM using a MagNA Pure 96 (Roche, Basel, Switzerland). SARS-CoV-2-RNA was detected by real-time RT-PCR confirming the presence of the viral E and RdRP genes (TIB molbiol, Berlin, Germany) or E and S genes (altona Diagnostics, Hamburg, Germany), respectively, using a LightCycler 480 or LightCycler 480 II (Roche, Basel, Switzerland).

This retrospective analysis was approved by the Ethical Review Board of the University Hospital of Cologne (#20-1089).

## Patient characteristics

**Figure 2:**
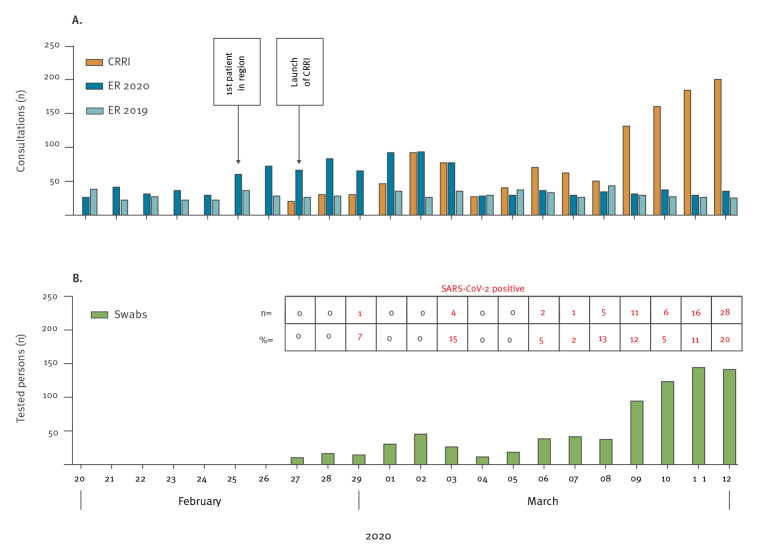
Consultations (A) and tested patients (B) at the COVID-19 rapid response infrastructure at University Hospital Cologne, North Rhine Westphalia, Germany, 27 February–12 March 2020

COVID-19: coronavirus disease; CRRI: COVID-19 rapid response infrastructure; ER: emergency room; SARS-CoV-2: severe acute respiratory syndrome coronavirus-2.

Within the period covered here, a total of 1,234 consultations of 1,166 patients occurred at the CRRI, which resulted in a marked decrease of suspected SARS-CoV-2 cases [5] that presented at the ER (Figure 2A). Consultations per day increased continuously in the second week, which was in line with the increase of total COVID-19 cases in Cologne and Germany (Supplementary Figure S1). A total of 177 (15%) of the individuals presenting at the ER were UHC employees for whom the CRRI provided a fast-track testing lane (Figure 1). Of UHC staff, 86 were symptomatic, 53 visited a risk area or had contact to a laboratory-confirmed COVID-19 case (n = 77). At least one risk of SARS-CoV-2 infection was present in 165 (93%) of them.

After risk assessment by physicians, 746 (64%) individuals (569 patients and 177 UHC staff) were tested for SARS-CoV-2 via nasopharyngeal swabs (Table) and 73 (10%) of them, including four UHC staff, tested positive for SARS-CoV-2 (Figure 2B); 2% of the UHC personnel that consulted the CRRI tested positive (Supplementary Figure S2). Notably, six of the 73 individuals testing positive for SARS-CoV-2 were asymptomatic. In exceptional cases, the test indication had been extended to persons who lived in the same household and who expressed the particular wish to be tested.

**Table:** Characteristics of SARS-CoV-2-tested individuals at the COVID-19 rapid response infrastructure at University Hospital Cologne and predictors for positive test results by univariable and multivariable logistic regression analysis, North Rhine Westphalia, Germany, 27 February–12 March 2020 (n = 746)

	SARS-CoV-2 positive n = 73				SARS-CoV-2 negative n = 673				Univariable regression^a^			Multivariable regression^a^		
	n	%	Median	IQR	n	%	Median	IQR	OR	95% CI	p value	OR	95% CI	p value
**Male**	45	62	NA	NA	320	48	NA	NA	1.78	1.09–2.91	0.023	1.85	1.00–3.42	0.05
**Age in years**	NA	NA	43	35–54	NA	NA	35	27–48	1.04	10.3–1.06	< 0.001	1.05	1.03–1.07	< 0.001
Risk factor														
Contact^b^	37	51	NA	NA	319	47	NA	NA	2.46	1.07–5.67	0.035	3.09	1.21–7.90	0.018
Area^c^	32	44	NA	NA	188	28	NA	NA	3.97	1.70–9.22	0.001	4.19	1.63–10.8	0.003
None	4	6	NA	NA	166	25	NA	NA	ref	ref	NA	ref	ref	NA
Symptoms^d,e^														
Yes	67	92	NA	NA	483	72	NA	NA	4.39	1.87–11.0	0.001	^d^	^d^	NA
Days since onset of symptoms^f^	NA	NA	3	1–5	NA	NA	4	2–7	0.89	0.81–0.97	0.007	0.87	0.78–0.96	0.008
Cough	54	74	NA	NA	353	53	NA	NA	2.58	1.49–4.45	0.001	1.73	0.79–3.89	0.173
Muscle or body aches	33	45	NA	NA	88	13	NA	NA	5.49	3.29–9.16	< 0.001	3.85	2.02–7.34	< 0.001
Fever	28	38	NA	NA	114	17	NA	NA	3.05	1.83–5.10	< 0.001	2.14	1.12–4.05	0.02
Headache	23	32	NA	NA	129	19	NA	NA	1.94	1.14–3.30	0.014	1.09	0.57–2.11	0.779
Sore throat	22	30	NA	NA	245	36	NA	NA	0.76	0.45–1.27	0.29	0.77	0.35–1.17	0.499
Rhinitis	20	27	NA	NA	240	36	NA	NA	0.69	0.39–1.17	0.161	0.47	0.21–1.02	0.054
Abnormal fatigue	7	9	NA	NA	58	9	NA	NA	1.13	0.49–2.56	0.78	0.61	0.23–1.60	0.316

CI: confidence interval; COVID-19: coronavirus disease; IQR: interquartile range; NA: Not applicable; OR: odds ratio; ref: reference category; SARS-CoV-2: severe acute respiratory syndrome coronavirus 2.

^a^ Uni- and multivariable logistic regression was performed. The final multivariable regression was performed using the enter methods (no cut-off) with variables for sex, age, risk for transmission, days since onset of symptoms, fever, cough, cold, sore throat, muscle or body aches, headache, and abnormal fatigue.

^b^ Contact to a laboratory-confirmed COVID-19 case in the last 14 days [5].

^c^ Visited an area at risk in the last 14 days [5].

^d^ Excluded due to multicollinearity.

^e^ Reference is no symptom for each category.

^f^ Missing for 51 individuals (n = 49 SARS-CoV-2 negative and n = 2 SARS-CoV-2 positive).

## Predictors for positive test results

We identified several independent predictors for positive test results by multivariable logistic regression analysis. These comprise a higher age (odds ratio (OR): 1.05; 95% confidence interval (CI): 1.03–1·07) a reported contact to an infected individual or a visit to an area at risk in the last 14 days (OR: 3.09; 95% CI: 1.21–7.90 and OR: 4.19; 95% CI: 1.63–10.79, respectively), a shorter time between onset of symptoms and testing (OR: 0.87; 95% CI: 0.78–0.96), fever > 38.5 °C (OR: 2.14; 95% CI: 1.12–4.05) and muscle or body aches (OR: 3.85; 95% CI: 2.02–7.34) as independent predictors for a positive SARS-CoV-2 testing (Table).

## Regular reviews to adjust risk evaluation

**Figure 3:**
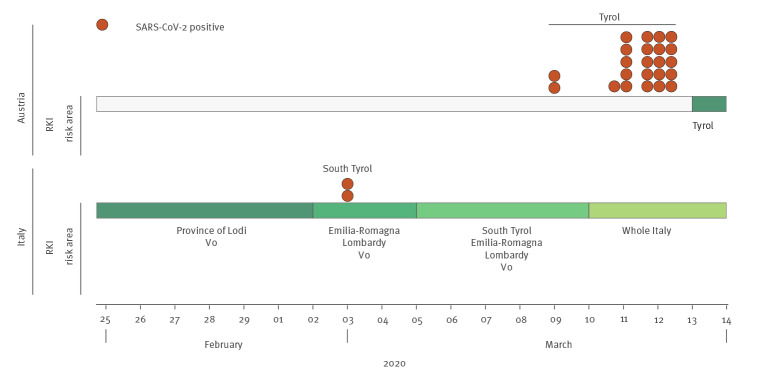
Early extension of risk areas at the COVID-19 rapid response infrastructure, University Hospital Cologne, Germany, February 2020

CRRI: COVID-19 rapid response infrastructure; RKI: Robert Koch Institute; SARS-CoV-2: severe acute respiratory syndrome coronavirus 2.

Seven days a week, a morning briefing was held where changes regarding the COVID-19 risk areas declared by the RKI or, if relevant, modifications of procedures, were discussed. Additionally, test results from previous days were reviewed and linked to individual patient travel anamnesis (e.g. symptomatic individuals who had travelled in regions bordering risk areas) media reports of other countries. By wider testing than per national recommendations, detection of potential new risk areas and clusters that were not yet covered by the recommendation of the RKI at that time, were identified. Early on, we defined two regions (South Tyrol Province, Italy and Tyrol Province, Austria) as risk areas, leading us to identify 25 SARS-CoV-2-positive cases that would not have received testing at the time of consultation if only the current national recommendations for risk areas would have been applied (Figure 3). Another six SARS-CoV-2-positive cases lacked any symptoms, which was a prerequisite for being categorised as an individual at risk by the RKI, based on the available evidence at the time. Thus, in total 31 of 73 (42%) laboratory-confirmed COVID-19 identified at UHC during the studied period would not have been tested following national guidelines.

Bars show changes in risk areas in Austria (upper bar) and Italy (lower bar) as defined by the German national public health institute (Robert Koch Institute (RKI)) over time. The respective RKI areas at risk are depicted below by bars which change colour when new areas were added (no area at risk was defined for Austria before 13 March 2020). Orange dots indicate the number of patients (one dot per patient) on each respective day that tested positive for SARS-CoV-2 due to the extended risk areas used for testing at the CRRI.

## Discussion

Germany is among the countries with the highest numbers of SARS-CoV-2 infections and with a high number of tests per capita in Europe [7]. There is a broad consensus that early diagnosis of infected individuals and their isolation is a cornerstone of controlling the spread of COVID-19 [8-11]. Thus, a rapid roll-out of testing capacities combined with triage and consultation by telephone in order to identify suspected cases, are crucial [12].

Here we describe the establishment of a CRRI and its impact on the management of large numbers of potentially SARS-CoV-2 infected patients at the ER of a modern tertiary-care hospital with more than 10,700 employees and a capacity of approximately 1,540 beds. We show that a dedicated facility for testing and managing patients can be rapidly operational. Such a facility allows for testing of large numbers of people at risk including hospital staff and facilitates the triage of attending patients with respiratory symptoms. Moreover, the inclusion of university hospital and public health staff seems particularly advantageous as it enables for example rapid contact tracing within 24 hours. Thus, a CRRI can serve as a template for further roll-out of testing sites into the surrounding region. While it was established and operational, mobile test stations or drive-in facilities could be set-up, solutions that require several days of installation until they are fully functional.

Daily data evaluation served to flexibly tailor procedures to the evolving situation and we were able to early identify two regions (Italian Tyrol and Austrian Tyrol) as risk areas. However, we did find ourselves in a conflict between case definitions by health authorities and case-based clinical reasoning. By applying the latter, we identified patients infected with SARS-CoV-2, who would not have been detected if solely the RKI recommendations had been applied. This information was then used to update the national guidelines on testing with regard to these risk areas.

The proportion of laboratory-confirmed COVID-19 cases older than 60 years of age was low (> 60 years: 6% (n = 45), Supplementary Figure S3) in our study population. The median age of patients testing positive in our cohort at the beginning of the pandemic was 43 years, which mirrored the situation of Germany as a whole, where the median age of COVID-19 cases was substantially lower than in other countries (Germany: 47 years; Italy: 63 years; China: 66 years) [13-15]. Moreover, the low median age could be explained by the fact that the majority of our cases were young travellers coming back from skiing holidays and our own hospital staff. In the meantime, the median age of COVID-19 cases in Germany increased to 50 years [16].

Predictors for SARS-CoV-2 positivity in our cases were in line with those identified by others [17-19]: older age (median age 43 vs 35), contact to a laboratory-confirmed COVID-19 case, visit to a risk area within 14 days before symptom onset, fever, muscle or body aches, and shorter duration between onset of symptoms and testing.

A main goal of the CRRI was to relieve the ER from a critically high patient load and to maintain its functionality. Here we were successful as shown by the discordant slopes of patient numbers in the ER and in the CRRI (Figure 2A). A triage system that allows care for individuals with no or with little symptoms separately from severely ill patients is important to prevent nosocomial transmission from low-risk to high-risk individuals. Timely on-site evaluation of positive cases regarding travel history or putative place of infection allowed for adaptation of test indications. We could show that testing criteria and definitions of high-risk areas published by national public health institutes may lag behind in a highly dynamic epidemic because of reporting delays, necessary validation steps and far-reaching implications.

In conclusion, we propose to establish dedicated facilities for testing and managing patients that comprise clinical and public health experts early in the course of a major epidemic as an important measure for prevention and mitigation.

## Supplementary Data

96000 Supplementary Figures
