# Supplementary material for: Rapid response infrastructure for pandemic preparedness in a tertiary care hospital: lessons learned from the COVID-19 outbreak in Cologne, Germany, February to March 2020
Source: Euro Surveill. 2020 May 28;25(21):2000531. doi: 10.2807/1560-7917.ES.2020.25.21.2000531 (PMC7268272; doi:10.2807/1560-7917.ES.2020.25.21.2000531)
Supplement: Supplementary Figures [file 2000531_LEHMANN_Supplementary_Figures.pdf]

Supplementary Material

Rapid response infrastructures for pandemic preparedness in tertiary care hospitals - lessons learned from the COVID-19 outbreak in Germany

This supplementary material is hosted by Eurosurveillance as supporting information alongside the article "Rapid response infrastructures for pandemic preparedness in tertiary care hospitals - lessons learned from the COVID-19 outbreak in Germany", on behalf of the authors, who remain responsible for the accuracy and appropriateness of the content. The same standards for ethics, copyright, attributions and permissions as for the article apply. Supplements are not edited by Eurosurveillance and the journal is not responsible for the maintenance of any links or email addresses provided therein.

Supplementary Figure S1

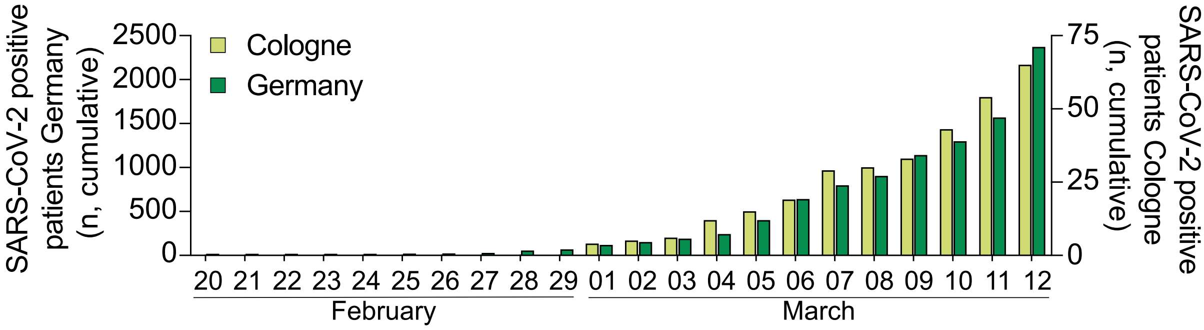

Cumulative SARS-CoV-2 positive tested patients of Cologne (light green) and Germany (dark green) as reported by the city of Cologne and the RKI, respectively.

Supplementary Figure S2

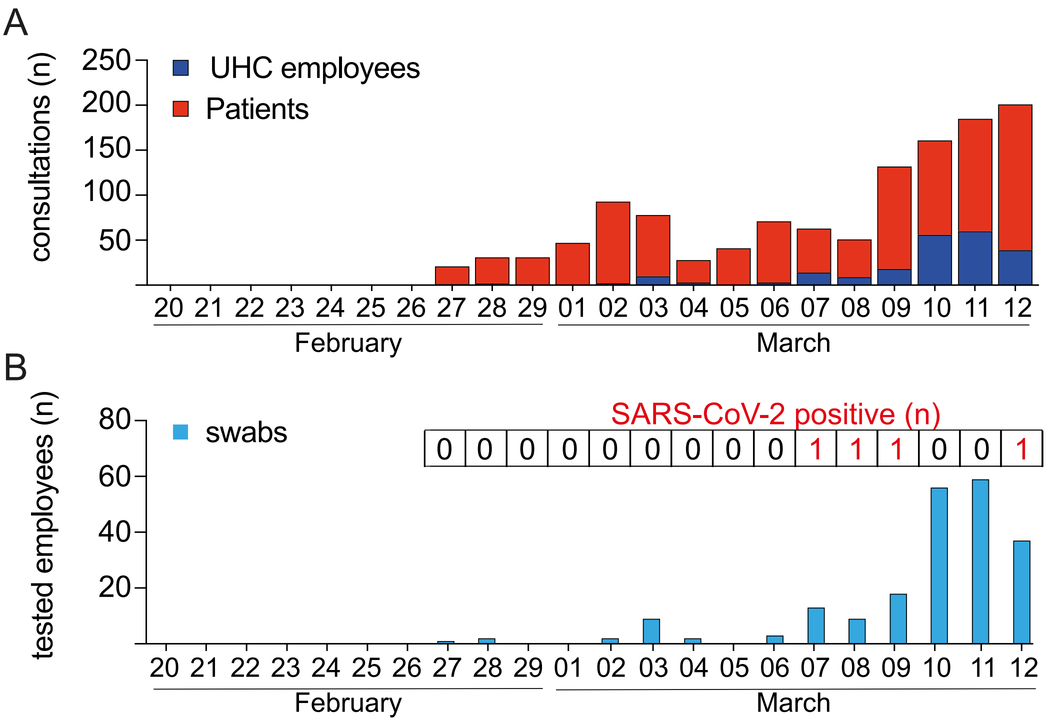

(A) Patients (red) and University Hospital of Cologne (UHC) employees (blue) consultations at the COVID-19 Rapid Response Infrastructure (CRRI). Bars show consultations to the CRRI per day, respectively. **(B)** Tested UHC employees at the CRRI per day. Bars show swabs taken per day which corresponds to patients tested per day. Table shows the SARS-CoV-2 positive tested UHC employees of each respective day.

Supplementary Figure S3

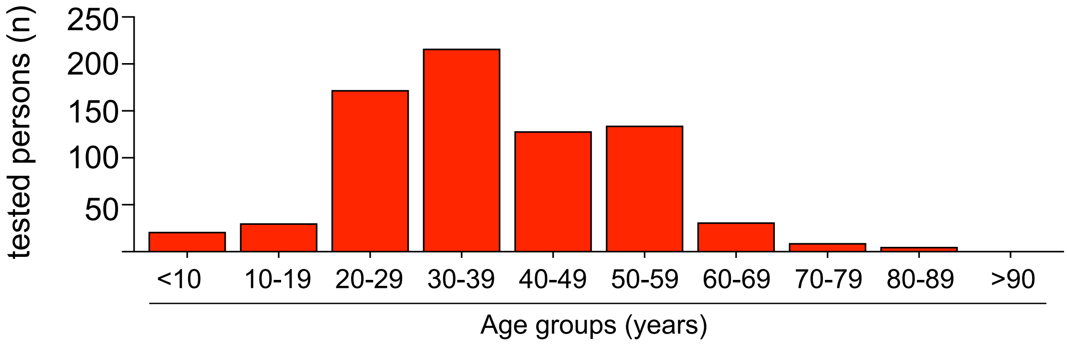

Bars show number of tested patients at the CRRI per respective age group.
